# Sensory‐motor network topology in multiple sclerosis: Structural connectivity analysis accounting for intrinsic density discrepancy

**DOI:** 10.1002/hbm.24989

**Published:** 2020-05-15

**Authors:** Simona Schiavi, Maria Petracca, Matteo Battocchio, Mohamed M. El Mendili, Swetha Paduri, Lazar Fleysher, Matilde Inglese, Alessandro Daducci

**Affiliations:** ^1^ Department of Computer Science University of Verona Verona Italy; ^2^ Department of Neuroscience, Rehabilitation, Ophthalmology, Genetics Maternal and Child Health (DINOGMI), University of Genoa Genova Italy; ^3^ Department of Neurology Icahn School of Medicine at Mount Sinai New York New York USA; ^4^ Department of Radiology Icahn School of Medicine at Mount Sinai New York New York USA; ^5^ Ospedale Policlinico San Martino IRCCS Genoa Italy

**Keywords:** COMMIT, diffusion MRI, graph theory, motor network, multiple sclerosis, structural connectivity, tractography

## Abstract

Graph theory and network modelling have been previously applied to characterize motor network structural topology in multiple sclerosis (MS). However, between‐group differences disclosed by graph analysis might be primarily driven by discrepancy in density, which is likely to be reduced in pathologic conditions as a consequence of macroscopic damage and fibre loss that may result in less streamlines properly traced. In this work, we employed the convex optimization modelling for microstructure informed tractography (COMMIT) framework, which, given a tractogram, estimates the actual contribution (or weight) of each streamline in order to optimally explain the diffusion magnetic resonance imaging signal, filtering out those that are implausible or not necessary. Then, we analysed the topology of this ‘COMMIT‐weighted sensory‐motor network’ in MS accounting for network density. By comparing with standard connectivity analysis, we also tested if abnormalities in network topology are still identifiable when focusing on more ‘quantitative’ network properties. We found that topology differences identified with standard tractography in MS seem to be mainly driven by density, which, in turn, is strongly influenced by the presence of lesions. We were able to identify a significant difference in density but also in network global and local properties when accounting for density discrepancy. Therefore, we believe that COMMIT may help characterize the structural organization in pathological conditions, allowing a fair comparison of connectomes which considers discrepancies in network density. Moreover, discrepancy‐corrected network properties are clinically meaningful and may help guide prognosis assessment and treatment choice.

## Graphical abstract

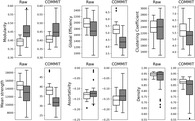

## 1 INTRODUCTION

Graph theory and network modelling have been applied to characterize structural motor network topology in multiple sclerosis (MS), demonstrating a reduced motor network efficiency through the quantification of structural damage in white matter (WM) bundles connecting pairs of cortical and subcortical grey matter (GM) regions (Pardini et al., 2015). More broadly, graph analysis of the structural connectome (Sporns, Tononi, & Kötter, 2005) (i.e., the set of white‐matter pathways between pairs of GM regions) has been successfully used to discriminate MS patients from healthy controls (HCs) and to classify MS clinical phenotypes (Kocevar et al., 2016; Li et al., 2013; Llufriu et al., 2017; Nigro et al., 2015). However, such between‐group differences may be primarily driven by discrepancy in network density (van Wijk, Stam, & Daffertshofer, 2010), which is likely to be reduced in pathologic conditions as a consequence of macroscopic damage and fibres loss. Thus, resulting in a less accurate tracking of streamlines (Ozturk et al., 2010). In the framework of graph analysis, methods such as the minimum spanning tree have been applied to account for differences in density, by reducing networks to a backbone structure insensitive to alterations in connection strength or linked density (Tewarie, van Dellen, Hillebrand, & Stam, 2015). An alternative and indirect way to deal with group differences in density is to extract connectivity metrics from an atlas of bundles built from healthy subjects keeping network density constant (Pagani et al., 2019). Tracing fibres in HC offers the additional advantage to avoid inaccuracy in tract reconstruction related to the presence of WM lesions. Therefore, in MS studies, tractography is often performed in the control group (or a subset of it), and the reconstructed tracts are subsequently registered to patients' data to derive the metrics of interest (Pagani et al., 2019; Pardini et al., 2015; Steenwijk et al., 2015). Although the underlying idea is the same, its implementation is slightly different in each of these works. Pagani et al. (2019) first coregistered the diffusion tensor images of HCs to the standard Montreal Neurological Institute (MNI) space, then they used the average of those data to perform tractography saving only the tracts connecting pairs of cortical areas with more than five streamlines as voxel maps. Finally, they registered all the remaining subjects to MNI space and they used the common tractogram to compute the individual connectomes. Pardini et al. (2015) instead performed tractography in each individual healthy subject's space and then registered the recovered track density images to the MNI space to create population‐averaged maps for each tracts of interest. They then coregistered these maps to each subject involved in the study to compute the connectomes. Finally Steenwijk et al. (Steenwijk et al., 2015) implemented a similar method of Pardini et al. (2015), but they computed for each subject and tract separately the average of weighted lesion volume and weighted average of fractional anisotropy (FA) in normal appearing WM. When tractography is conducted directly in MS patients, an FA threshold is set during fibre reconstruction and a minimum number of fibres are selected to define single bundles in order to reduce the risk of false‐positive connections (Nigro et al., 2015; Shu et al., 2011). The shortcoming of this approach is the drastic reduction in reconstructed fibres, especially in those bundles that are rich in crossing fibres (Sinke et al., 2018). More recently, a spherical‐deconvolution‐informed filtering of tractograms (SIFT; R. E. Smith, Tournier, Calamante, & Connelly, 2013) has been employed to reduce reconstruction bias and improve biological plausibility (Koubiyr et al., 2019), but the accuracy of SIFT application to pathological brains is still under debate (Zalesky, Sarwar, & Ramamohanarao, 2020).

Furthermore, the characterization of the structural connectome in MS has to take into account the impact of WM lesions on connectivity which is usually assessed through correlation analysis between graph metrics and lesion loads (He et al., 2009; Romascano et al., 2015). A more specific disconnection analysis can also be conducted, quantifying dedicated graph measures that estimate the indirect, compensatory connections between two regions developed after the transection of the direct connection between them (Li et al., 2013). More recently, the impact of macroscopic lesions on structural connectivity was modelled by assuming transection of all fibres passing through WM lesions (Pagani et al., 2019).

Finally, the quantification of the connection strength in structural connectomes is an open issue. Typically, the connection strength between each pair of grey‐matter regions is ‘quantified’ by counting the number of streamlines connecting them, that is, streamline count, but this approach is not quantitative (Jones, Knösche, & Turner, 2013). Microstructure‐informed tractography (Daducci, Dal Palu, Descoteaux, & Thiran, 2016) was recently proposed as a means to improve the estimation of structural connectivity by combining tractography with local microstructural features of the tissue and fitting the actual contributions of the streamlines to the measured diffusion magnetic resonance imaging (MRI) data. These contributions do not allow to estimate the microscopical fibre count, but this approach has the potential to provide a more ‘physically quantitative’ assessment of the connectivity than the simple streamline count. In fact, as the contributions of the streamlines (or weights) are estimated such that they explain the diffusion MRI data, and the connectivity is ‘physically quantified’ based on these weights. This possibility to extract more ‘quantitative’ metrics from the reconstructed connectomes may allow for a fair comparison of network properties despite density discrepancies. However, to the best of our knowledge, this approach has never been proposed in clinical studies.

In this proof of concept study, we investigated the topology of the ‘physically quantitative’ sensory‐motor network (SMN) (i.e., the network whose weights are estimated through microstructure informed tractography) in MS using the convex optimization modelling for microstructure‐informed tractography (COMMIT) (Daducci, Dal Palu, Lemkaddem, & Thiran, 2013, 2015). COMMIT allows the tracking of fibres within WM lesions and removes the ones deemed implausible according to the chosen microstructural property only after reconstruction. The goal of this study was to test if abnormalities in network topology are still identifiable when focusing on more ‘quantitative’ network properties. We focused on patients with progressive MS (PMS), who present the highest lesion loads, atrophy degree and, presumably, density reduction among MS clinical phenotypes. Specifically, we evaluated if (a) COMMIT can improve the detection of differences in structural connectome density between MS patients and HC compared to the raw connectome; (b) differences in network density affect between‐group comparisons of connectome properties; (c) WM lesions and GM atrophy influence connectome properties; and (d) SMN network properties are related to clinical disability.

## 2 MATERIALS AND METHODS

### 2.1 Subjects

Forty‐two patients with PMS (22 primary and 20 secondary progressive 28F, mean age 51.4 ± 11.4 years, mean disease duration 15.6 ± 13.3 years) and 24 HC (11F, mean age 50.3 ± 8.5 years) were prospectively enrolled. Inclusion criteria for patients with MS were age between 18 and 70 years, MS diagnosis fulfilling the revised McDonald criteria (Polman et al., 2011) and Expanded Disability Status Scale (EDSS) score ≤7.0. Exclusion criteria were coexistence of any major systemic condition, diagnosis of psychiatric disorders, contraindications to undergo an MRI scan, pregnancy, history of head trauma, alcoholism, drug addiction, or neurological disorders other than MS. Clinical examination, performed within 1 week from the MRI scan, included EDSS, timed 25‐foot walk test (T25FWT) and 9‐hole peg test (9HPT). Written informed consent was obtained from all participants before the beginning of the study procedures, according to the Declaration of Helsinki. The protocol was approved by the Institutional Review Board of the Icahn School of Medicine at Mount Sinai.

### 2.2 MRI acquisition

All subjects underwent MRI on a Siemens Skyra 3T scanner (Siemens, Erlangen, Germany) with a 32‐channel head coil. The MRI protocol included the following sequences: axial T2‐weighted 3D (repetition time [TR]: 8000 ms, echo time [TE]: 95 ms, spatial resolution 0.5 × 0.5 × 3.0 mm^3^); sagittal T1‐weighted 3D magnetization‐prepared rapid gradient echo (TR/TE: 3000/2.47 ms, inversion time [TI]: 1000 ms, spatial resolution 0.8 × 0.8 × 0.8 mm^3^; generalized autocalibrating partially parallel acquisitions with acceleration factor *R* = 2); twice‐refocused spin echo echo‐planar imaging sequence for diffusion MRI with *b* values of 1,000 and 2,000 s/mm^2^ and 30 directions each (repeated twice), in addition to *b* = 0 images (TR/TE: 4,700/100 ms, flip angle 80°, spatial resolution 1.8x1.8x2 mm^3^).

### 2.3 Lesion and cortical segmentations

Quantification of T2‐hyperintense and T1‐hypointense lesion volume was performed in each patient by a single experienced observer unaware of subject identity, employing a segmentation technique based on user‐supervised local thresholding (Jim 7.0, Xinapse System, Leicester, UK, http://www.xinapse.com) as described in Petracca et al. (2018). The corresponding T1 images were then accordingly filled using T1‐hypointense lesion mask and FMRIB software library (FSL) (https://fsl.fmrib.ox.ac.uk).

**Figure 1:**
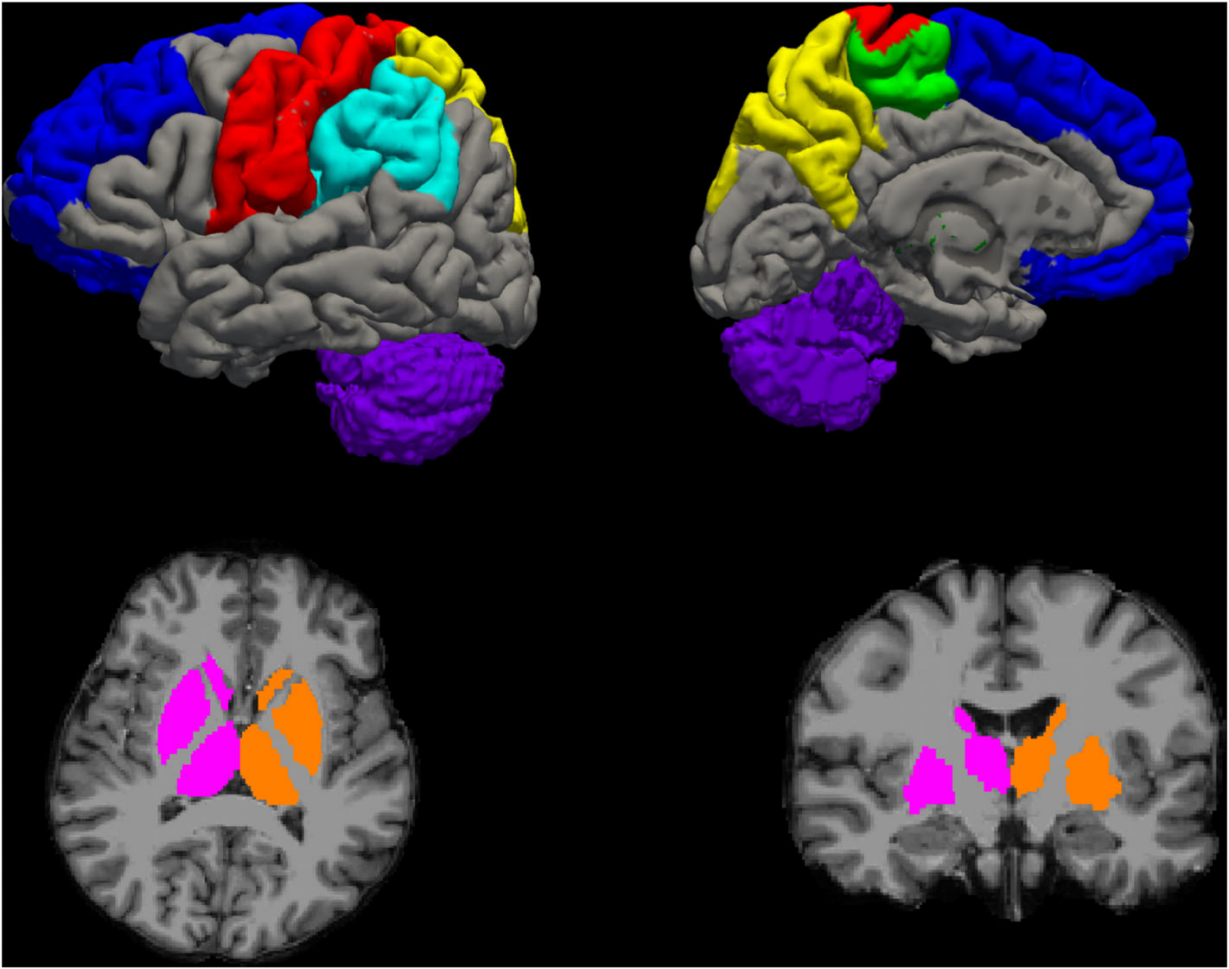
Motor network hubs used in our analysis in a representative healthy subject. The primary sensory‐motor cortex (S‐M1) is shown in red; the secondary motor cortex (M2) in green; the secondary sensory cortex (S2) in light blue; the posterior associative sensory cortex (AS Sens C) in yellow; the prefrontal cortex (PFC) in blue; the deep grey matter (Deep GM) in pink (for the right hemisphere) and orange (for the left hemisphere) and the cerebellum in purple

For all subjects, we processed T1‐filled images with FreeSurfer (http://surfer.nmr.mgh.harvard.edu) and we automatically segmented them (Fischl et al., 2002; Fischl et al., 2004) using the standard Desikan–Killiany atlas (Desikan et al., 2006) which allowed obtaining a cortical parcellation in 85 regions of interest (ROIs). From this parcellation, we retrieved the nodes of the motor network comparing FreeSurfer ROIs and the Harvard–Oxford cortical and subcortical structural atlas included in FSL (S. M. Smith et al., 2004). In particular, the primary sensory‐motor cortex (S‐M1) was defined by the post central and precentral gyrus ROIs; the secondary motor cortex (M2) by the paracentral gyrus ROI; the secondary sensory cortex (S2) by the supramarginal gyrus; the posterior associative sensory cortex (AS Sens C) by the precuneus and superior parietal gyrus ROIs; the prefrontal cortex (PFC) by the lateral orbitofrontal, medial orbitofrontal, rostral middle frontal and superior frontal ROIs; the deep GM (Deep GM) by the union of the thalamus, caudate, putamen and pallidum ROIs acting as relay for projection tracts and, finally, the cerebellum (cerebellum) as itself. The obtained nodes for one of the healthy subjects included in our analyses are shown in Figure 1.

SMN GM fraction (GMF) was computed as the sum of the volumes of all the above‐listed areas divided by intracranial volume.

### 2.4 Diffusion MRI processing

Diffusion MR images were corrected for motion and eddy currents (Andersson & Sotiropoulos, 2016) using FSL. To perform whole brain anatomically constrained tractography (R. E. Smith, Tournier, Calamante, & Connelly, 2012), we first coregistered the T1 and diffusion images using FMRIB's linear image registration tool (FLIRT) (Jenkinson, Bannister, Brady, & Smith, 2002) of FSL with boundary‐based cost function (Greve & Fischl, 2009). Then we computed the fibre orientation distribution functions using the multishell multitissue‐constrained spherical deconvolution approach (Jeurissen, Tournier, Dhollander, Connelly, & Sijbers, 2014; Tournier, Calamante, & Connelly, 2007) and generated 1 million streamlines using the iFOD2 (Tournier, Calamante, & Connelly, 2010) tractography algorithm implemented in MRtrix (http://www.mrtrix.org). In light of the discussion in Zalesky et al (2020), we processed the resulting tractograms using the COMMIT (Daducci et al., 2013, 2015) with stick and zeppelin ball model (Alexander et al., 2010). COMMIT is a powerful framework that allows to decompose a signal in contributions coming from different compartments. The main assumption of the framework is that the contribution of a streamline is constant along its path, while the remaining components can be different in each voxel. In this case, we applied COMMIT to diffusion MR signal and we decomposed the signal in intra‐axonal, extra‐axonal and isotropic contributions according to the stick and zeppelin ball model (Alexander et al., 2010). Indeed, with this model, we imposed that the intra‐axonal diffusion signal was constant along each tract and (when needed) we indirectly accounted for the presence of free water due to a lesion with the zeppelin and ball compartments.

**Figure 2:**
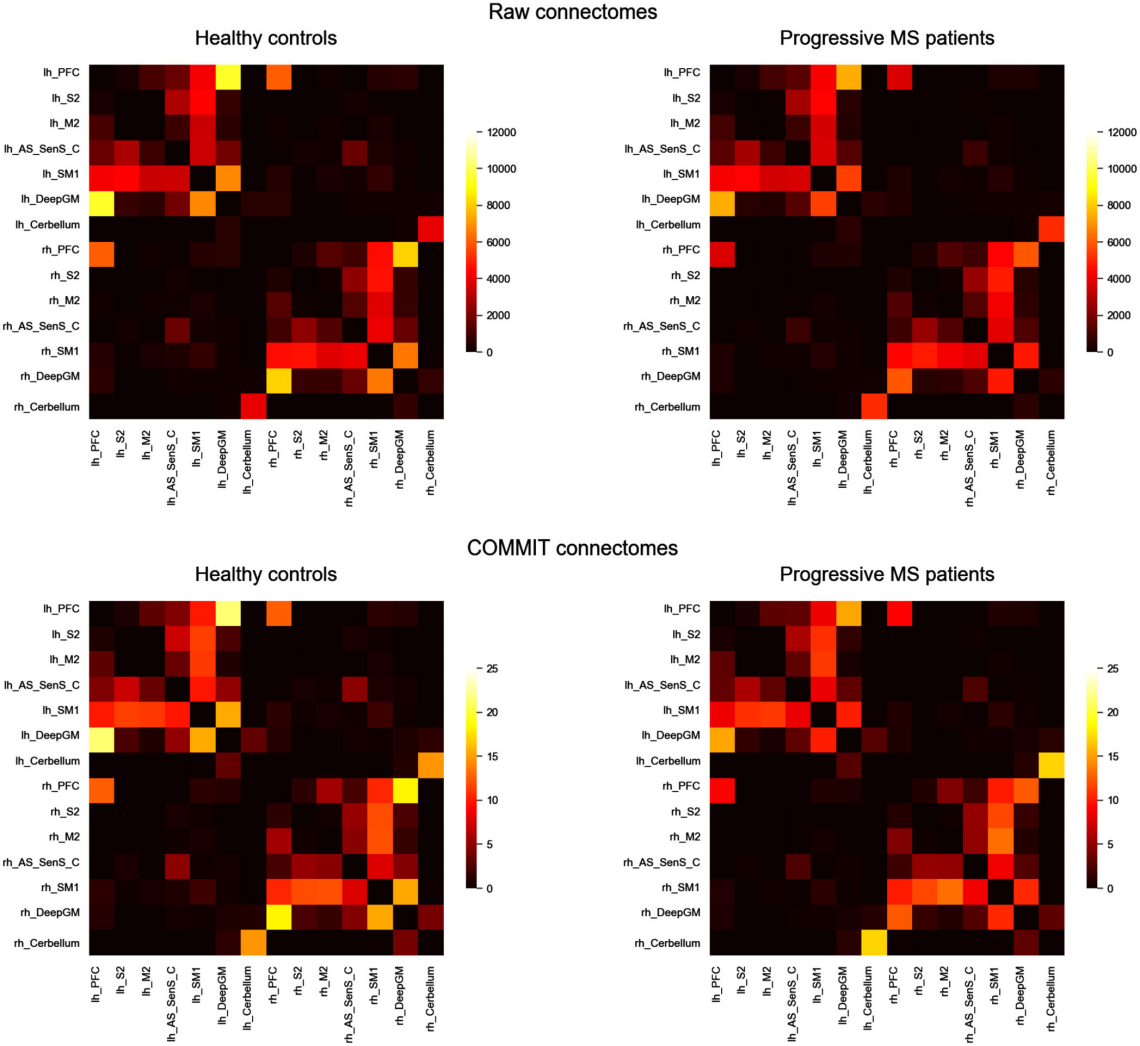
Matrix representation of the connectomes obtained with the two different methods: counting the number of streamlines connecting two pairs of grey matter regions (top); or assigning the quantitative measures obtained with COMMIT (bottom). For both method we report the average connectomes obtained for the two groups of subjects: healthy controls (left) and PMS patients (right). In both cases (raw and COMMIT), the pattern of connections is similar, but while in the upper case the information contained in the connectomes is nonquantitative, in the bottom ones it represents the intra‐axonal signal fraction associated to each connection. We also observe that some interhemispheric connections present in the raw connectomes disappear after the application of COMMIT. COMMIT, convex optimization modelling for microstructure informed tractography; PMS, progressive multiple sclerosis

Finally, for each subject, both the raw (i.e., obtained using the number of streamlines as entries) and the COMMIT‐weighted connectomes (i.e., obtained using COMMIT weights as entries) were built using the motor network parcellation described above (Figure 2). As entries (*a*_*ij*_) of COMMIT‐derived matrices, we used the weighted average intra‐axonal signal contribution of each bundle:

$$a_{\mathrm{ij}}=\frac{\sum_{k=1}^{N_{\mathrm{ij}}}x_{\mathrm{ij}}^{k}∙l_{k}\;}{\frac{\sum_{k=1}^{N_{\mathrm{ij}}}l_{k}}{N_{\mathrm{ij}}}},$$

where *i* and *j* are the indices of ROIs connected by the bundle, *N*_*ij*_ is bundle's number of streamlines, $x_{\mathrm{ij}}^{k}$ is the weight of the streamline *k* obtained by COMMIT and *l*_*k*_ its length. In this way, each entry contained the total signal fraction associated to the bundle, which was given by the weighted average of the streamline contribution (obtained with COMMIT) multiplied by its length and divided by the average length of the bundle.

In light of the recent results showed in Buchanan et al. (2020), in Data S1, we also report additional results obtained by thresholding the number of streamlines in the raw connectomes according to two widely used techniques: proportional and consistency thresholding. For further details, we recommend readers to refer Data S1.

### 2.5 Graph analysis

As it was done in previous works (Pagani et al., 2019; Pardini et al., 2015; Steenwijk et al., 2015), for each subject we computed six global network measures from the obtained connectomes using the brain connectivity toolbox (Rubinov & Sporns, 2010): *modularity* (reflecting the segregation of the network), *global efficiency* (corresponding to the average inverse shortest path length in the network and inversely related to the characteristic path length), *clustering coefficient* (reflecting the degree to which the nodes tend to cluster together), *mean strength* (corresponding to the average of all the nodal strengths, where the nodal strength is the sum of the weights of links connected to the node), *assortativity* (reflecting if nodes tend to be connected to other nodes with similar strengths) and *density* (corresponding to the fraction of present connections to possible connections). For each node of the subjects' connectome we also computed local efficiency and nodal strength to investigate which node of the SMN was more affected by the disease.

### 2.6 Statistical analysis

All analyses were performed using Statistical Package for Social Science (SPSS V.25.0).

Between‐group comparisons were performed via analysis of covariance analysis, entering age and gender as covariates. In order to assess differences in density estimation related to the application of COMMIT, we performed between‐group comparisons both on results from raw connectomes and COMMIT‐weighted connectomes and repeated the analysis entering density as additional covariate.

The relationship between network global properties, T2 lesion load and GM atrophy were tested via partial correlation accounting for age and gender.

The relationship between network properties and clinical disability was tested with stepwise regression models, entering age and gender in the first block and network global/local properties in the second block.

Results were considered significant for *p* < .05 (Bonferroni corrected <0.008 for global properties [0.05/6 as the number of network global properties considered]; Bonferroni corrected <0.003 for local properties [0.05/14 as the number of nodes considered]).

## 3 RESULTS

### 3.1 Between‐group differences in connectome properties

#### 3.1.1 Raw connectomes

Mean values and *SDs* of the global network metrics are reported in Table 1. After Bonferroni correction for multiple comparisons, modularity, global efficiency and mean strength were significantly different between the two groups of subjects when accounting for age and sex. When controlling also for density only the difference in modularity was still present. Of note, no significant differences in density were identified between the two groups.

**Table 1:** Global graph metrics of HCs and PMS patients computed on the raw connectomes

	HC (*n* = 24)	PMS (*n* = 42)	*p* ^a^	*p* ^b^
Modularity	0.39 ± 0.03	0.46 ± 0.06	**<.001**	**<.001**
Global efficiency	1997.23 ± 242.51	1,716.31 ± 379.93	**.003**	.024
Clustering coefficient	2,376.28 ± 281.07	2,340.14 ± 385.03	.958	.327
Mean strength	14,726.32 ± 1,742.35	12,701.39 ± 2,682.28	**.002**	.017
Assortativity	−0.13 ± 0.02	−0.12 ± 0.03	.113	.208
Density	0.94 ± 0.02	0.91 ± 0.08	.055	–

Abbreviations: HCs, healthy controls; PMS, progressive multiple sclerosis.

*Note:* All values are expressed as mean ± *SD*; ANCOVA age and gender corrected (*p* ^a^), ANCOVA age, gender and density corrected (*p* ^b^). Statistically significant *p* values after Bonferroni correction are highlighted in bold.

Mean values and *SDs* of the local network metrics are reported in Table 2 (strength) and Table 3 (efficiency). After Bonferroni correction for multiple comparisons, significant differences were identified in five nodes in terms of strength and in nine nodes in terms of efficiency between the two groups of subjects when accounting for age and sex. When controlling also for density, significant differences were still identified in two nodes in terms of strength and in six nodes in terms of efficiency.

**Table 2:** Nodes strength of HCs and PMS patients computed on the raw connectomes

	Side	HC (*n* = 24)	PMS (*n* = 42)	*p* ^a^	*p* ^b^
PFC	R	22,763.00 ± 3,061.27	17,223.40 ± 5,448.31	**<.001**	**<.001**
	L	24,068.79 ± 3,684.92	19,031.62 ± 6,314.12	**.001**	.010
S2	R	8,730.58 ± 1,326.41	8,490.48 ± 1832.01	.685	.417
	L	8,847.83 ± 1,428.36	8,494.86 ± 1819.53	.508	.651
M2	R	8,164.50 ± 1,553.38	7,556.55 ± 1,558.34	.129	.428
	L	6,648.67 ± 1,451.18	6,142.43 ± 1,421.49	.201	.452
As Sens C	R	12,782.87 ± 2,542.15	10,918.00 ± 3,097.91	.029	.216
	L	13,511.79 ± 2027.72	11,186.31 ± 3,014.71	**.002**	.018
S‐M1	R	25,710.50 ± 3,185.54	23,091.50 ± 4,319.22	.025	.215
	L	24,168.08 ± 3,993.54	22,589.05 ± 4,580.20	.219	.866
Deep GM	R	19,671.92 ± 3,032.35	14,175.50 ± 5,019.85	**<.001**	**<.001**
	L	21,266.62 ± 3,421.53	16,600.62 ± 5,764.56	**.001**	.004
Cerebellum	R	5,024.42 ± 2,200.44	6,222.33 ± 2,177.42	.037	.059
	L	4,808.92 ± 2,198.79	6,096.78 ± 2,301.40	.037	.061

Abbreviations: AS Sens C, posterior associative sensory cortex; Deep GM, deep grey matter; HCs, healthy controls; M2, secondary motor cortex; S‐M1, sensory‐motor cortex; S2, secondary sensory cortex; PFC, prefrontal cortex; PMS, progressive multiple sclerosis.

*Note:* All values are expressed as mean ± *SD*; ANCOVA age and gender corrected (*p* ^a^), ANCOVA age, gender and density corrected (*p* ^b^). Statistically significant *p* values after Bonferroni correction are highlighted in bold.

**Table 3:** Nodes efficiency of HC and PMS patients computed on the raw connectomes

	Side	HC (*n* = 24)	PMS (*n* = 42)	*p* ^a^	*p* ^b^
PFC	R	709.40 ± 103.54	546.64 ± 171.56	**<.001**	**.001**
	L	707.44 ± 124.48	556.60 ± 190.01	**.002**	.012
S2	R	401.98 ± 53.94	385.79 ± 100.87	.398	.073
	L	379.30 ± 62.29	370.41 ± 89.42	.766	.274
M2	R	434.33 ± 76.70	352.43 ± 83,17	**<.001**	**.002**
	L	401.06 ± 67.52	335.49 ± 88.80	**.002**	.006
As Sens C	R	620.74 ± 111.40	479.05 ± 131.44	**<.001**	**<.001**
	L	656.76 ± 110.77	492.80 ± 122.97	**<.001**	**<.001**
S‐M1	R	839.94 ± 128.53	674.43 ± 172.13	**<.001**	**.001**
	L	783.84 ± 138.19	654.77 ± 171.33	.004	.028
Deep GM	R	646.60 ± 109.95	498.40 ± 159.60	**<.001**	**.001**
	L	658.61 ± 107.49	537.86 ± 161.56	**.002**	.014
Cerebellum	R	163.01 ± 61.32	153.46 ± 50.50	.608	.911
	L	130.99 ± 52.71	129.97 ± 44.51	.977	.602

Abbreviations: AS Sens C, posterior associative sensory cortex; Deep GM, deep grey matter; HCs, healthy controls; M2, secondary motor cortex; S‐M1, sensory‐motor cortex; S2, secondary sensory cortex; PFC, prefrontal cortex; PMS, progressive multiple sclerosis.

*Note:* All values are expressed as mean ± *SD*; ANCOVA age and gender corrected (*p* ^a^), ANCOVA age, gender and density corrected (*p* ^b^). Statistically significant *p* values after Bonferroni correction are highlighted in bold.

#### 3.1.2 COMMIT‐weighted connectomes

Mean values and *SDs* of the global network metrics are reported in Table 4. After Bonferroni correction for multiple comparisons, all the explored metrics, except the clustering coefficient, were significantly different between the two groups of subjects when controlling for age and sex. When controlling also for density the difference in assortativity disappeared.

**Table 4:** Global graph metrics of HCs and PMS patients on COMMIT‐weighted connectomes

	HC (*n* = 24)	PMS (*n* = 42)	*p* ^a^	*p* ^b^
Modularity	0.41 ± 0.02	0.46 ± 0.05	**<.001**	**.005**
Global efficiency	5.27 ± 0.62	4.35 ± 0.52	**<.001**	**<.001**
Clustering coefficient	5.67 ± 0.81	5.18 ± 0.70	.024	.025
Mean strength	38.15 ± 4.22	31.64 ± 3.88	**<.001**	**<.001**
Assortativity	−0.16 ± 0.03	−0.13 ± 0.04	**.006**	.188
Density	0.88 ± 0.26	0.82 ± 0.09	**.004**	–

Abbreviations: COMMIT, convex optimization modelling for microstructure informed tractography; HCs, healthy controls; PMS, progressive multiple sclerosis.

*Note:* All values are expressed as mean ± *SD*. Raw *p* values from the post hoc test to compare subject groups in terms of the network metrics are reported in the last two columns; *p* ^a^ comparison controlling for age and sex; *p* ^b^, comparison controlling for age, sex and density. Statistically significant *p* values after Bonferroni correction are highlighted in bold.

Mean values and *SDs* of the local network metrics are reported in Table 5 (strength) and Table 6 (efficiency). After Bonferroni correction for multiple comparisons, significant differences were identified in six nodes in terms of strength and in seven nodes in terms of efficiency between the two groups of subjects when accounting for age and sex. When controlling also for density significant differences were still identified in the same nodes in terms of strength and in seven nodes in terms of efficiency.

**Table 5:** Nodes strength of HCs and PMS patients computed on COMMIT‐weighted connectomes

	Side	HC (*n* = 24)	PMS (*n* = 42)	*p* ^a^	*p* ^b^
PFC	R	53.16 ± 9.33	40.86 ± 8.78	**<.001**	**<.001**
	L	54.88 ± 7.22	42.81 ± 10.57	**<.001**	**.001**
S2	R	22.19 ± 5.71	19.53 ± 4.47	.064	.235
	L	23.20 ± 4.95	19.21 ± 4.66	**.002**	.011
M2	R	25.54 ± 6.42	24.68 ± 6.77	.587	.500
	L	19.69 ± 5.61	18.60 ± 4.99	.425	.107
As Sens C	R	31.19 ± 4.43	26.55 ± 6.54	.005	.079
	L	36.77 ± 7.37	27.43 ± 6.34	**<.001**	**<.001**
S‐M1	R	62.95 ± 11.77	56.30 ± 9.03	.027	.129
	L	62.66 ± 13.90	52.03 ± 8.32	**<.001**	**.002**
Deep GM	R	49.71 ± 6.63	34.08 ± 10. 30	**<.001**	**<.001**
	L	52.55 ± 14.48	36.69 ± 9.90	**<.001**	**<.001**
Cerebellum	R	20.35 ± 7.87	22.26 ± 7.73	.232	.698
	L	19.30 ± 7.30	21.88 ± 7.72	.174	.676

Abbreviations: AS Sens C, posterior associative sensory cortex; COMMIT, convex optimization modelling for microstructure informed tractography; Deep GM, deep grey matter; HCs, healthy controls; M2, secondary motor cortex; S‐M1, sensory‐motor cortex; S2, secondary sensory cortex; PFC, prefrontal cortex; PMS, progressive multiple sclerosis.

*Note:* All values are expressed as mean ± *SD*; ANCOVA age and gender corrected (*p* ^a^), ANCOVA age, gender and density corrected (*p* ^b^). Statistically significant *p* values after Bonferroni correction are highlighted in bold.

**Table 6:** Nodes efficiency of HCs and PMS patients computed on COMMIT‐weighted connectomes

	Side	HC (*n* = 24)	PMS (*n* = 42)	*p* ^a^	*p* ^b^
PFC	R	1.84 ± 0.34	1.48 ± 0.23	**<.001**	**<.001**
	L	1.71 ± 0.28	1.43 ± 0.31	**.001**	.022
S2	R	1.12 ± 0.22	1.23 ± 0.52	.349	.233
	L	1.10 ± 0.20	1.10 ± 0.49	.918	.007
M2	R	1.25 ± 0.25	1.19 ± 0.36	.456	.022
	L	1.12 ± 0.20	1.19 ± 0.57	.531	.060
As Sens C	R	1.59 ± 0.18	1.41 ± 0.35	.026	**.001**
	L	1.82 ± 0.26	1.46 ± 0.45	**.001**	**<.001**
S‐M1	R	2.12 ± 0.32	1.78 ± 0.41	**.001**	**.001**
	L	2.12 ± 0.36	1.69 ± 0.36	**<.001**	**<.001**
Deep GM	R	1.72 ± 0.21	1.37 ± 0.23	**<.001**	**<.001**
	L	1.72 ± 0.28	1.37 ± 0.32	**<.001**	**<.001**
Cerebellum	R	0.76 ± 0.26	0.67 ± 0.18	.095	.080
	L	0.79 ± 0.26	0.70 ± 0.20	.094	.140

*Note:* All values are expressed as mean ± *SD*; ANCOVA age and gender corrected (*p* ^a^), ANCOVA age, gender and density corrected (*p* ^b^). Statistically significant *p* values after Bonferroni correction are highlighted in bold.

### 3.2 Relationship between raw connectome global properties, WM lesions and GM atrophy

Accounting for age and gender, significant correlations were identified between T2 lesion volume and global efficiency (*r* = −.655, *p* < .0001), clustering coefficient (*r* = −.469, *p* = .002), modularity (*r* = .640, *p* < .0001), density (*r* = −.696, *p* < .0001), mean strength (*r* = −.630, *p* < .0001). No correlations were identified between SMN GMF and global metrics.

### 3.3 Relationship between COMMIT‐weighted connectome global properties, WM lesions and GM atrophy

Accounting for age and gender, significant correlations were identified between T2 lesion volume and global efficiency (*r* = −.431, *p* = .005), modularity (*r* = .507, *p* = .001) and density (*r* = −.738, *p* < .0001) as well as between SMN GMF and mean strength (*r* = .425, *p* = .006).

### 3.4 Clinical impact of raw connectome abnormalities

The models including demographic variables and network global properties accounted for 40% of variance in 9HPT scores (for density *R* ^2^ = .40, *p* = .001,*β* = −.57, *p* = .003) and 32% of variance in 25FWT (for density *R* ^2^ = .32, *p* = .004, *β* = −.51, *p* = .001; for assortativity *R* ^2^ = .32, *p* = .004, *β* = .51, *p* = .001). No significant results were yielded by the model including demographic variables and node local properties.

### 3.5 Clinical impact of COMMIT‐weighted connectome abnormalities

The models including demographic variables and network global properties accounted for 27% to 35% of variance in 9HPT scores (for modularity *R* ^2^ = .27, *p* = .018, *β* = .45, *p* = .007; for density *R* ^2^ = .35, *p* = .003, *β* = −.53, *p* = .001). The model including demographic variables and node local properties accounted for 58% of variance in 9HPT scores (for right PFC local efficiency *R* ^2^ = .58, *p* = .008, *β* = −.53, *p* = .003) and 66% of variance in T25FWT scores (for associative sensory cortex local efficiency *R* ^2^ = .66, *p* = .001, *β* = 1.12, *p* < .001).

## 4 DISCUSSION

Notwithstanding all previous efforts in investigating structural connectivity and disconnection in MS, in this study we propose a methodological approach—COMMIT—that accounts for the presence of lesions and fibres loss and provides a means to directly compare connectomes with different density.

**Figure 3:**
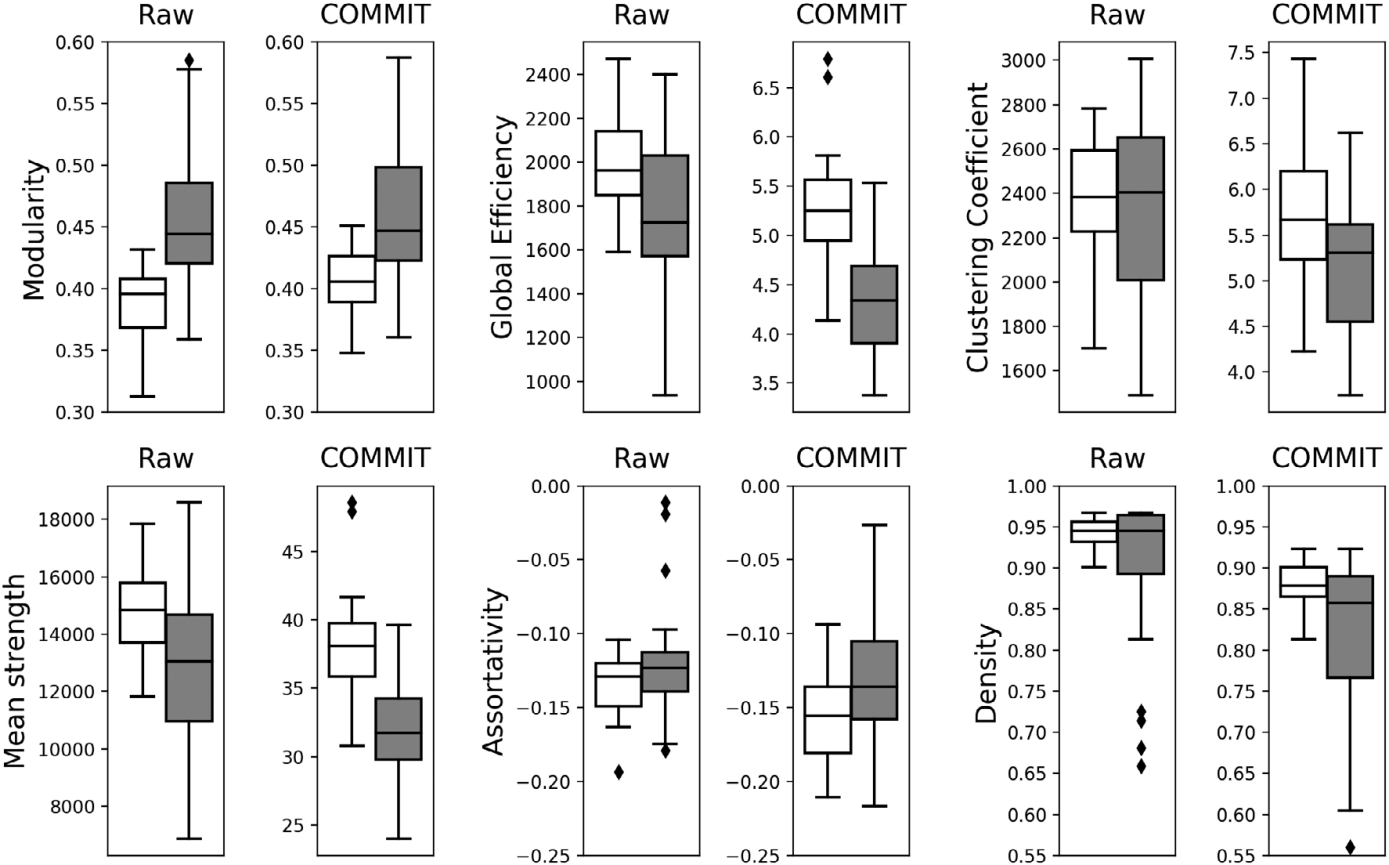
Boxplots showing the differences in global network measures between HCs (white) and PMS patients (grey) for both raw and COMMIT tractograms. We observe that after the application of COMMIT the differences between HC and PMS patients are more pronounced. Also, the presence of outliers is often mitigated when COMMIT is applied. COMMIT, convex optimization modelling for microstructure informed tractography; HCs, healthy controls; PMS, progressive multiple sclerosis

**Figure 4:**
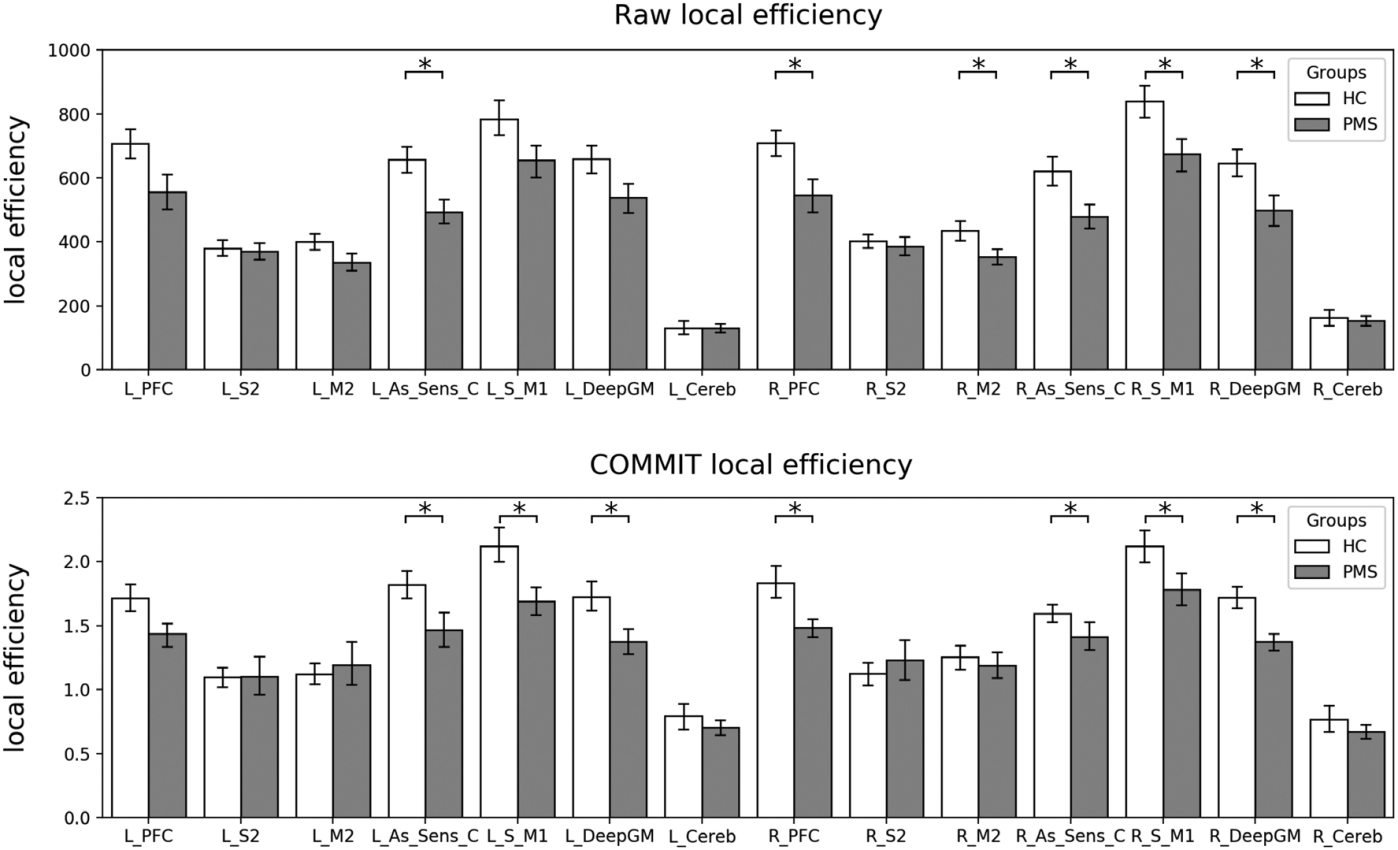
Barplot showing the local efficiency of all the hubs of the motor network for both raw and COMMIT connectomes. The statistically significant differences between HCs in white and PMS patients in grey and accounting for discrepancies in age, sex and density are marked with an asterisk. COMMIT, convex optimization modelling for microstructure informed tractography; HCs, healthy controls; PMS, progressive multiple sclerosis

**Figure 5:**
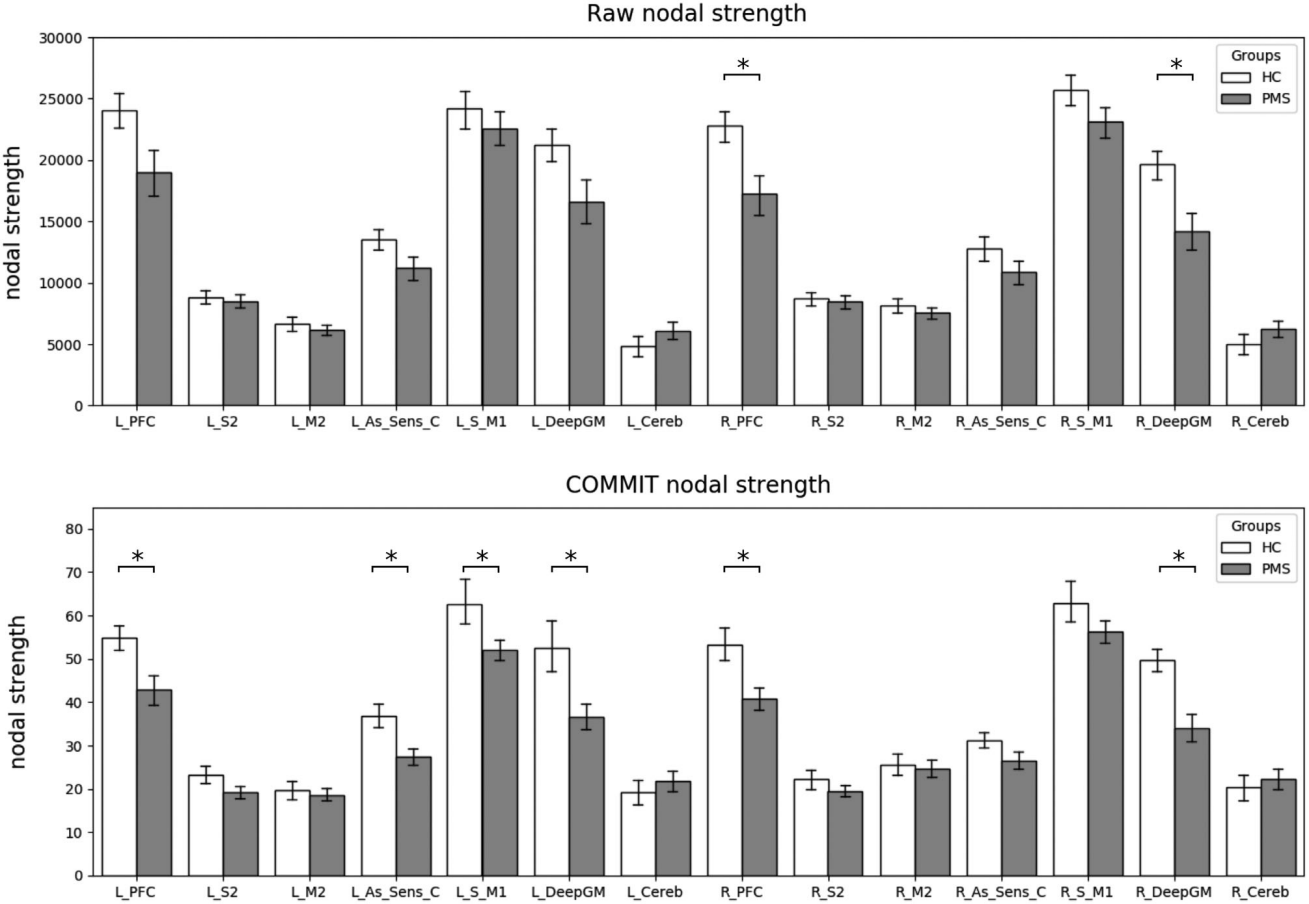
Barplot showing the strength of all the nodes of the motor network for both raw and COMMIT tractograms. The statistically significant differences between HCs in white and PMS patients in grey and accounting for discrepancies in age, sex and density are marked with an asterisk. With the application of COMMIT differences in the left associative sensory cortex, sensory‐motor and right deep grey matter strength appears. COMMIT, convex optimization modelling for microstructure informed tractography; HCs, healthy controls; PMS, progressive multiple sclerosis

Thanks to its capability of decomposing the intrinsic signal contribution of each streamline in the tractogram, COMMIT may represent an effective method to cope with density discrepancies between healthy subjects and patients. The main idea behind this method is to assume that one (or more) microstructure feature does not vary along the length of a tract and therefore it is possible to effectively estimate its value for the entire tract (rather than only voxel‐wise). This estimation is done simultaneously for all the streamlines by fitting them to a map related to the selected microstructure feature. If only diffusion‐weighted magnetic resonance imaging data are available, it is reasonable to assume that the intra‐axonal diffusion signal is constant along the tract and COMMIT uses any predefined microstructural model to estimate it. Similarly to what was recently found in Lipp et al. (2019), using the recently introduced multishell multitissue spherical deconvolution (Jeurissen et al., 2014) and the probabilistic algorithm (Tournier et al., 2010) to generate streamlines, we were able to propagate the tracking also inside MS lesions to build the input tractograms. We then applied COMMIT to decide if a streamline passing through a lesion is essential to explain the signal or not and consequently keeps or discards it to construct the final tractogram. In the present work, we employed as microstructural model the stick and zeppelin ball model (Alexander et al., 2010) which indirectly accounts for the presence of free water due to a lesion with the zeppelin and ball compartments. Finally, to construct the COMMIT‐weighted connectomes, we chose not to use the traditional number of streamlines connecting two cortical regions of interest (streamlines count), which was shown not to be quantitative (Jones et al., 2013). Conversely, we considered the more informative total signal fraction associated to the bundle, which is given by the weighted average of the streamline contribution (obtained with COMMIT) multiplied by its length and divided by the average length of the bundle. This approach offers two main advantages. First, by forcing fibre tracking within lesions and subsequently filtering them according to the signal preservation along the streamline, COMMIT retains in the tractogram only fibres whose microstructure is not irredeemably damaged by lesions or subtle inflammatory/neurodegenerative processes ongoing in the normal appearing WM (Lassmann, 2018). Thus, producing a weighted network composed by ‘healthy’ and partly damaged fibres whose signal is not irreversibly compromised and can be fitted with a stick. As a consequence of COMMIT's filtering, in the COMMIT‐weighted connectomes, we observed a reduction in density in comparison with the raw connectomes both in patients and controls (Figure 3). A number of implausible connections, related to tractography intrinsic limitations, as well as the fact that our control group presumably presented age‐related subtle WM abnormalities, were removed in HCs. As expected though, the number of implausible connections removed in patients was even higher, which explains why differences in terms of density between patients and controls became apparent only after COMMIT application. Second, by giving the possibility to compare more ‘quantitative’ metrics rather than measures derived from the nonquantitative streamline number (Jones et al., 2013), COMMIT offers the possibility to assess differences in network properties beyond changes driven by density discrepancy. This is supported by the results of our between‐group comparison, which shows that, while topology differences identified with standard tractography were mainly driven by density, differences in global and local properties derived from the COMMIT‐weighted connectomes were insensitive to density correction (Figures 3, 4, 5). Finally, it is worth highlighting that although COMMIT estimates the actual weight of the edges in the network by fitting the corresponding streamlines to the white‐matter signal, normalization may still be required to account for ROI size differences in the chosen parcellation (Sotiropoulos & Zalesky, 2019). In fact, larger ROIs may be connected with more streamlines simply because of their size. Note, however, that this applies to raw and COMMIT‐weighted connectomes alike, and hence it does not bias our results. Future studies will investigate the possibility to use COMMIT to account also for this aspect.

Differences in connectome global properties estimated after COMMIT application suggest that also the COMMIT‐weighted connectome presents the topology abnormalities previously described in MS (Kocevar et al., 2016; Li et al., 2013; Llufriu et al., 2017; Nigro et al., 2015; Pardini et al., 2015). Indeed, the COMMIT‐weighted SMN was less efficient, more dispersed and weaker in MS than in HC, supporting the notion that also seemingly intact connections are not sufficient to preserve brain structure. As COMMIT retains also connections partly affected by WM lesions, WM bundles entered in the COMMIT‐weighted connectome still suffer the consequences of smouldering inflammation, axonal and neuronal damage within focal lesions, and periventricular damage sustained by detrimental soluble factors (Lassmann, 2018). Fibres damage and loss above a certain threshold could eventually leave a vulnerable structure, not able to sustain efficient network function. Assortativity was the only network property still affected by density after COMMIT application, suggesting that nodes' connection strength in the COMMIT‐weighted connectome depends on the presence of preserved connections. The strong link between density and assortativity is also highlighted by their comparable predictive power on clinical disability. Locally, strength and efficiency were decreased in the PFC, primary sensory‐motor areas, associative sensitive cortex and deep GM, confirming the diffuse involvement of cortical and deep GM regions reported in the progressive phenotypes (Eshaghi et al., 2018) (Figures 4 and 5). COMMIT‐weighted SMN global properties showed strong to moderate associations with WM lesion load and atrophy, confirming that brain topological organization is related to the accrual of macrostructural damage (Pagani et al., 2019), with lesion load playing a predominant role in PMS (Steenwijk et al., 2015). Of note though, raw SMN global properties showed even stronger relationships with WM lesion load, once again supporting the notion that network properties derived from raw connectomes are substantially influenced by the presence of lesions. On the other hand, the effects of atrophy were not detectable, possibly because of the dominant influence of WM lesion load itself. As per the clinical impact of network topology, raw connectomes properties were not predictive of clinical status, while among COMMMIT‐weighted connectomes properties the main role was played by nodes’ local efficiency, which predicted a large amount of variance in motor disability. PFC efficiency was particularly relevant for manual dexterity performance, highlighting the importance of motor planning for the execution of fine motor movements, while efficiency of associative sensory cortex was significantly correlated with the ambulation performance. Interestingly, it seems that the efficiency of integrative rather than primary areas is particularly relevant for clinical function preservation within the weighted connectome, highlighting the compensatory role of these regions in advanced disease stages.

## 5 CONCLUSIONS

Topology differences identified with standard tractography in MS seem to be mainly driven by density, which, in turn, is strongly influenced by the presence of lesions, suggesting caution when interpreting between‐group differences in connectome properties. Moving from a qualitative towards a more ‘quantitative’ appraisal of the brain structural connectome, COMMIT application allowed the identification of a significant difference in density between patients and HC and the exploration of network topology in the COMMIT‐weighted connectome. Differences observed in network global and local properties suggest that preserved connections undergo a topological reorganization in MS. Within such reorganization of the brain connectome, decreased local efficiency in key areas of the SMN represent the most relevant correlates of motor disability. Based on these results, we believe that COMMIT may help characterize the topological organization of structural networks in pathological conditions, allowing a fair comparison of connectomes which takes into account discrepancies in network density. More importantly, our study shows that discrepancy‐corrected network properties are clinically meaningful and, therefore, may help guide prognosis assessment and treatment choice.

## Supporting information


**Data S1**: Results using state‐of‐the‐art thresholding methods
**Table S1**: Global graph metrics of healthy controls (HC) and progressive MS (PMS) patients computed on the raw connectomes after the application of proportional thresholding. All values are expressed as mean standard deviation. Analysis of covariance (ANCOVA) age and gender corrected (*p* ^a^), ANCOVA age, gender and density corrected (*p* ^b^). Statistically significant *p*‐values after Bonferroni correction are highlighted in bold
**Table S2**: Nodes strength of healthy controls (HC) and progressive MS (PMS) patients computed on the raw connectomes after the application of proportional thresholding. All values are expressed as mean standard deviation. ANCOVA age and gender corrected (*p* ^a^), ANCOVA age, gender and density corrected (*p* ^b^). Statistically significant p‐values after Bonferroni correction are highlighted in bold
**Table S3**: Nodes efficiency of healthy controls (HC) and progressive MS (PMS) patients computed on the raw connectomes after the application of proportional thresholding. All values are expressed as mean standard deviation. ANCOVA age and gender corrected (*p* ^a^), ANCOVA age, gender and density corrected (*p* ^b^). Statistically significant p‐values after Bonferroni correction are highlighted in bold
**Table S4**: Global graph metrics of healthy controls (HC) and progressive MS (PMS) patients computed on the raw connectomes after the application of consistency thresholding. All values are expressed as mean standard deviation except for the density which is imposed to be 30% by the method. ANCOVA age and gender corrected p are reported in the last column. Statistically significant *p*‐values after Bonferroni correction are highlighted in bold
**Table S5**: Nodes strength of healthy controls (HC) and progressive MS (PMS) patients computed on the raw connectomes after the application of consistency thresholding. All values are expressed as mean standard deviation. ANCOVA age and gender corrected p are reported in the last column. Statistically significant *p*‐values after Bonferroni correction are highlighted in bold
**Table S6**: Nodes efficiency of healthy controls (HC) and progressive MS (PMS) patients computed on the raw connectomes after the application of consistency thresholding. All values are expressed as mean standard deviation. ANCOVA age and gender corrected *p* are reported in the last column. Statistically significant *p*‐values after Bonferroni correction are highlighted in bold

**Figure S1** Comparison of the behaviour of the 91 connections in raw and COMMIT‐weighted connectomes for both healthy controls (HC) and progressive multiple sclerosis patients (PMS). The overall behaviour of the connections’ strength shows a high correlation between the methods but looking at individual connections we see how COMMIT is able to enlarge or shrink the differences between the two groups of subjects.

## Data Availability

The brain MRI images used were obtained from the Mount Sinai Hospital of New York and could available from the corresponding author upon reasonable request.
