# Supplementary material for: Sensory‐motor network topology in multiple sclerosis: Structural connectivity analysis accounting for intrinsic density discrepancy
Source: Hum Brain Mapp. 2020 May 15;41(11):2951–63. doi: 10.1002/hbm.24989 (PMC7336144; doi:10.1002/hbm.24989)
Supplement: Supplementary file 1 — Data S1: Results using state‐of‐the‐art thresholding methods Table S1: Global graph metrics of healthy controls (HC) and progressive MS (PMS) patients computed on the raw connectomes after the application of proportional thresholding. All values are expressed as mean standard deviation. Analysis of covariance (ANCOVA) age and gender corrected (p a), ANCOVA age, gender and density corrected (p b). Statistically significant p‐values after Bonferroni correction are highlighted in bold Table S2: Nodes strength of healthy controls (HC) and progressive MS (PMS) patients computed on the raw connectomes after the application of proportional thresholding. All values are expressed as mean standard deviation. ANCOVA age and gender corrected (p a), ANCOVA age, gender and density corrected (p b). Statistically significant p‐values after Bonferroni correction are highlighted in bold Table S3: Nodes efficiency of healthy controls (HC) and progressive MS (PMS) patients computed on the raw connectomes after the application of proportional thresholding. All values are expressed as mean standard deviation. ANCOVA age and gender corrected (p a), ANCOVA age, gender and density corrected (p b). Statistically significant p‐values after Bonferroni correction are highlighted in bold Table S4: Global graph metrics of healthy controls (HC) and progressive MS (PMS) patients computed on the raw connectomes after the application of consistency thresholding. All values are expressed as mean standard deviation except for the density which is imposed to be 30% by the method. ANCOVA age and gender corrected p are reported in the last column. Statistically significant p‐values after Bonferroni correction are highlighted in bold Table S5: Nodes strength of healthy controls (HC) and progressive MS (PMS) patients computed on the raw connectomes after the application of consistency thresholding. All values are expressed as mean standard deviation. ANCOVA age and gender corrected p are reported in the las [file HBM-41-2951-s001.docx]

Supplementary Information

To make the differences between raw and COMMIT-weighted connectomes clearer, in the supplementary Figure S1 we compare the value of each connection in the raw connectomes (first row) and the COMMIT-weighted connectomes (second row), separately for HC and PMS patients. The connections are labeled from 0 to 90 and correspond to the upper diagonal entries of the symmetric connectivity matrix ordered by rows from left to right. We highlight that the raw and COMMIT-weighted connectomes are correlated for HC and PMS patients separately. This is because both contain measures that reflect the structural connectivity but looking at the individual connections, we can appreciate the effect of COMMIT. While assigning a physically quantitative meaning to the structural connectivity, what the framework does is to enlarge or to shrink the difference between HC and PMS in each connection, based on the fit of the input tractogram to the data. Therefore, for some connections even if the number of streamlines is higher in the PMS than in HC, by fitting the streamlines to the actual signal COMMIT can overturn the behavior and obtain that the strength of the connection is indeed higher in HC than PMS.

As a complementary experiment, we report the results obtained by thresholding the raw connectomes with two popular and widely used techniques: proportional and consistency thresholding. Based on the meta-analysis reported in (Buchanan et al., 2020), for the proportional thresholding we analysed only the connections present in at least 50% of the subjects enrolled in our study and the results are reported in Table S1-S3. For the consistency thresholding instead, for each connectome we retained only the 30% strongest connections and the results are reported in Table S4-S6. In our small networks (only 14x14 connections), the effect of proportional thresholding is barely visible. Indeed, all the results are comparable with the one obtained for the raw connectomes. Instead, after applying consistency thresholding while the global measures become comparable with the one obtained with COMMIT-connectomes, the local measures are hugely impacted by this type of thresholding. Indeed, since out network where already small by construction, keeping only 30% of the connections discards too many bundles of interest. Results obtained with this methodology suggest that this type of thresholding might be accurate when considering the whole brain network but not when only a smaller sub-network is under investigation.

# References

Buchanan, C. R., Bastin, M. E., Ritchie, S. J., Liewald, D. C., Madole, J. W., Tucker-Drob, E. M., Deary, I. J., & Cox, S. R. (2020). The effect of network thresholding and weighting on structural brain networks in the UK Biobank. *NeuroImage*, *211*, 116443. https://doi.org/https://doi.org/10.1016/j.neuroimage.2019.116443


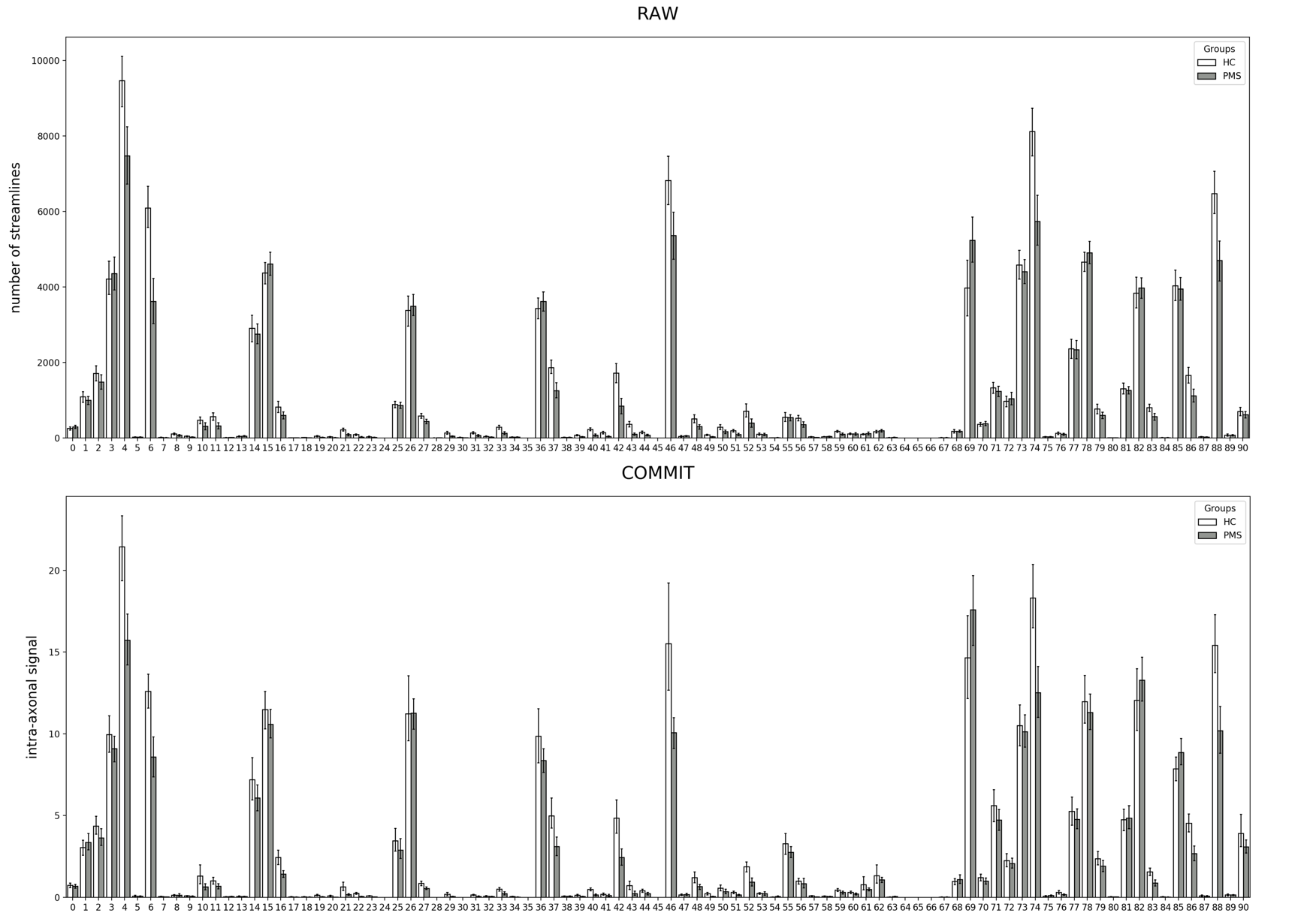


Figure S1: Comparison of the behaviour of the 91 connections in raw and COMMIT-weighted connectomes for both healthy controls (HC) and progressive multiple sclerosis patients (PMS). The overall behaviour of the connections’ strength shows a high correlation between the methods but looking at individual connections we see how COMMIT is able to enlarge or shrink the differences between the two groups of subjects.

|  | HC (n=24) | PMS (n=42) | p^a^ | p^b^ |
| --- | --- | --- | --- | --- |
| Modularity | 0.35 ± 0.03 | 0.43 ± 0.08 | **<0.001** | **<0.001** |
| Global Efficiency | 2934.52 ± 353.87 | 2513.19 ± 555.62 | **0.002** | 0.017 |
| Clusterig Coefficient | 3796.00 ± 437.87 | 3758.21 ± 636.02 | 0.936 | 0.295 |
| Mean Strength | 22089.48 ± 2613.53 | 19052.08 ± 4023.43 | **0.002** | 0.017 |
| Assortativity | -0.12 ± 0.02 | -0.11 ± 0.02 | 0.052 | 0.110 |
| Density | 0.93 ± 0.02 | 0.91 ± 0.08 | 0.055 | - |

Table S1: Global graph metrics of healthy controls (HC) and progressive MS (PMS) patients computed on the raw connectomes after the application of proportional thresholding. All values are expressed as mean standard deviation. ANCOVA age and gender corrected (p^a^), ANCOVA age, gender and density corrected (p^b^). Statistically significant p-values after Bonferroni correction are highlighted in bold.

|  | Side | HC (n=24) | PMS (n=42) | p^a^ | p^b^ |
| --- | --- | --- | --- | --- | --- |
| PFC | R | 38161.75 ± 5050.30 | 30060.83 ± 8767.79 | **<0.001** | **0.001** |
|  | L | 48137.58 ± 7369.85 | 38063.24 ± 12628.23 | **0.001** | 0.010 |
| S2 | R | 16664.83 ± 2630.00 | 16439.88 ± 3481.98 | 0.915 | 0.269 |
|  | L | 17444.67 ± 2824.87 | 16689.55 ± 3570.68 | 0.468 | 0.704 |
| M2 | R | 14116.71 ± 2879.46 | 13368.60 ± 2711.38 | 0.297 | 0.735 |
|  | L | 12161.25 ± 2791.40 | 11234.36 ± 2661.55 | 0.204 | 0.466 |
| As Sens C | R | 18505.71 ± 3899.99 | 16009.45 ± 4422.85 | 0.05 | 0.317 |
|  | L | 21515.92 ± 3487.09 | 17270.88 ± 4875.03 | **0.001** | **0.005** |
| S – M1 | R | 32260.21 ± 4256.16 | 27868.62 ± 5830.01 | **0.004** | 0.032 |
|  | L | 32937.83 ± 5847.19 | 29099.64 ± 6963.76 | 0.031 | 0.217 |
| Deep GM | R | 20375.04 ± 3152.99 | 14792.52 ± 5151.07 | **<0.001** | **<0.001** |
|  | L | 22969.88 ± 3777.25 | 18068.60 ± 6309.20 | **0.001** | **0.006** |
| Cerebellum | R | 5024.42 ± 2200.44 | 6222.33 ± 2177.42 | 0.037 | 0.059 |
|  | L | 8976.96 ± 4164.99 | 11540.64 ± 4386.41 | 0.031 | 0.056 |

Table S2: Nodes strength of healthy controls (HC) and progressive MS (PMS) patients computed on the raw connectomes after the application of proportional thresholding. All values are expressed as mean standard deviation. ANCOVA age and gender corrected (p^a^), ANCOVA age, gender and density corrected (p^b^). Statistically significant p-values after Bonferroni correction are highlighted in bold.

|  | Side | HC (n=24) | PMS (n=42) | p^a^ | p^b^ |
| --- | --- | --- | --- | --- | --- |
| PFC | R | 1023.28 ± 149.37 | 788.39 247.503 | **<0.001** | **0.001** |
|  | L | 1021.18 ± 179.56 | 803.29 274.287 | **0.002** | 0.012 |
| S2 | R | 580.15 ± 77.87 | 556.82 145.789 | 0.398 | 0.073 |
|  | L | 547.22 ± 89.89 | 534.33 129.027 | 0.764 | 0.272 |
| M2 | R | 626.92 ± 110.98 | 508.56 120.042 | **<0.001** | **0.002** |
|  | L | 578.54 ± 97.65 | 483.83 128.145 | **0.002** | **0.006** |
| As Sens C | R | 895.12 ± 160.85 | 690.80 ± 189.95 | **<0.001** | **<0.001** |
|  | L | 946.96 ± 159.60 | 710.27 ± 177.31 | **<0.001** | **<0.001** |
| S – M1 | R | 1213.98 ± 185.18 | 973.78 ± 248.61 | **<0.001** | **0.001** |
|  | L | 1130.92 ± 199.45 | 945.43 ± 247.24 | **0.004** | 0.029 |
| Deep GM | R | 932.60 ± 158.62 | 718.92 ± 230.37 | **<0.001** | **0.001** |
|  | L | 950.85 ± 155.11 | 776.13 ± 233.41 | **0.002** | 0.013 |
| Cerebellum | R | 234.99 ± 88.31 | 221.23 ± 72.83 | 0.608 | 0.909 |
|  | L | 188.92 ± 76.03 | 187.43 ± 64.19 | 0.978 | 0.602 |

Table S3: Nodes efficiency of healthy controls (HC) and progressive MS (PMS) patients computed on the raw connectomes after the application of proportional thresholding. All values are expressed as mean standard deviation. ANCOVA age and gender corrected (p^a^), ANCOVA age, gender and density corrected (p^b^). Statistically significant p-values after Bonferroni correction are highlighted in bold.

|  | HC (n=24) | PMS (n=42) | p |
| --- | --- | --- | --- |
| Modularity | 0.43 ± 0.03 | 0.48 ± 0.05 | **<0.001** |
| Global Efficiency | 100.65 ± 13.17 | 86.72 ± 18.92 | **0.004** |
| Clusterig Coeff | 140.12 ± 18.54 | 137.967857 26.31 | 0.949 |
| Mean Strength | 696.16 ± 77.87 | 614.34 ± 119.01 | **0.006** |
| Assortativity | 0.02 ± 0.06 | -0.03 ± 0.09 | 0.010 |
| Density | 0.30 | 0.30 | - |

Table S4: Global graph metrics of healthy controls (HC) and progressive MS (PMS) patients computed on the raw connectomes after the application of consistency thresholding. All values are expressed as mean standard deviation except for the density which is imposed to be 30% by the method. ANCOVA age and gender corrected p are reported in the last column. Statistically significant p-values after Bonferroni correction are highlighted in bold.

|  | Side | HC (n=24) | PMS (n=42) | p |
| --- | --- | --- | --- | --- |
| PFC | R | 1235.61 ± 176.51 | 987.39 ± 285.30 | **<0.001** |
|  | L | 1680.78 ± 263.84 | 1330.16 ± 438.68 | **0.001** |
| S2 | R | 559.75 ± 101.71 | 571.85 ± 113.47 | 0.555 |
|  | L | 541.42 ± 92.70 | 536.84 ± 113.61 | 0.972 |
| M2 | R | 368.11 ± 95.63 | 367.58 ± 86.79 | 0.912 |
|  | L | 367.47 ± 100.61 | 380.10 ± 94.39 | 0.569 |
| As Sens C | R | 653.28 ± 144.41 | 569.94 ± 163.60 | 0.085 |
|  | L | 675.09 ± 104.86 | 563.98 ± 154.50 | 0.004 |
| S – M1 | R | 1002.28 ± 124.34 | 889.24 ± 170.91 | 0.013 |
|  | L | 1024.16 ± 175.38 | 938.39 ± 197.69 | 0.095 |
| Deep GM | R | 601.34 ± 90.83 | 434.35 ± 152.18 | **<0.001** |
|  | L | 641.46 ± 112.82 | 510.27 ± 171.35 | **0.001** |
| Cerebellum | R | 144.07 ± 72.04 | 182.35 ± 68.20 | 0.037 |
|  | L | 251.40 ±125.83 | 338.25 ± 136.32 | 0.018 |

Table S5: Nodes strength of healthy controls (HC) and progressive MS (PMS) patients computed on the raw connectomes after the application of consistency thresholding. All values are expressed as mean standard deviation. ANCOVA age and gender corrected p are reported in the last column. Statistically significant p-values after Bonferroni correction are highlighted in bold.

|  | Side | HC | PMS | p |
| --- | --- | --- | --- | --- |
| PFC | R | 95.43 ± 30.33 | 76.22 ± 25.24 | 0.013 |
|  | L | 95.77 ± 23.64 | 83.88 ± 25.98 | 0.065 |
| S2 | R | 162.41 ± 35.30 | 150.43 ± 53.67 | 0.458 |
|  | L | 156.80 ± 34.43 | 160.71 ± 45.85 | 0.509 |
| M2 | R | 107.17 ± 13.63 | 98.07 ± 18.79 | 0.076 |
|  | L | 118.16 ± 28.98 | 109.69 ± 23.84 | 0.273 |
| As Sens C | R | 72.11 ± 10.37 | 77.40 ± 16.12 | 0.096 |
|  | L | 74.43 ± 12.12 | 77.81 ± 17.20 | 0.324 |
| S – M1 | R | 128.03 ± 24.87 | 121.81 ± 25.83 | 0.454 |
|  | L | 126.26 ± 26.52 | 123.60 ± 28.77 | 0.847 |
| Deep GM | R | 109.33 ± 30.42 | 91.00 ± 37.83 | 0.035 |
|  | L | 176.15 ± 39.64 | 135.80 ± 68.70 | 0.017 |
| Cerebellum | R | 0.00 ± 0.00 | 0.27 ± 1.77 | 0.313 |
|  | L | 0.00 ± 0.00 | 0.82 ± 5.30 | 0.313 |

Table S6: Nodes efficiency of healthy controls (HC) and progressive MS (PMS) patients computed on the raw connectomes after the application of consistency thresholding. All values are expressed as mean standard deviation. ANCOVA age and gender corrected p are reported in the last column. Statistically significant p-values after Bonferroni correction are highlighted in bold.
