# Supplementary figures and images for: Sensory‐motor network topology in multiple sclerosis: Structural connectivity analysis accounting for intrinsic density discrepancy
Source: Hum Brain Mapp. 2020 May 15;41(11):2951–63. doi: 10.1002/hbm.24989 (PMC7336144; doi:10.1002/hbm.24989)

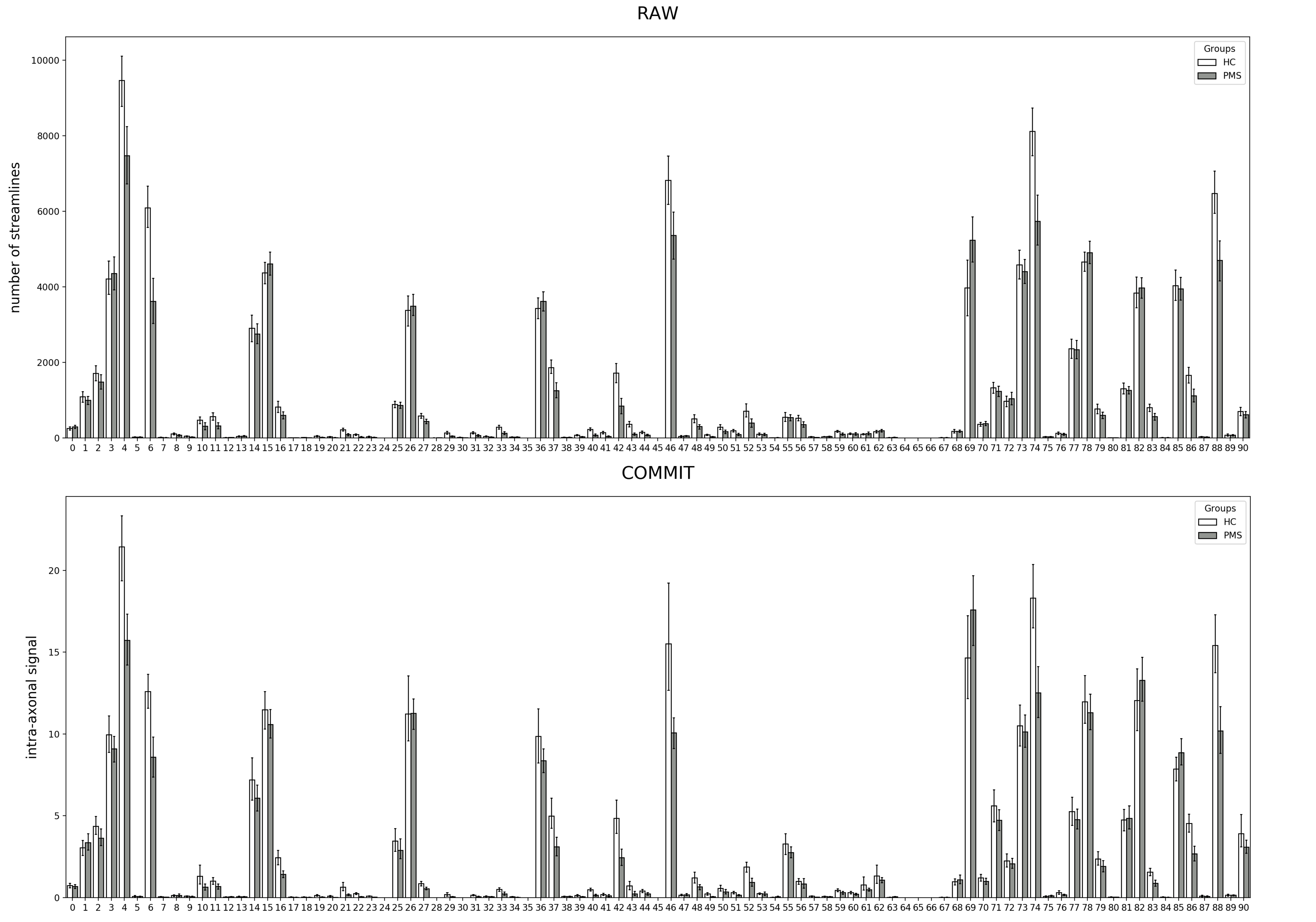

Supplement: Supplementary file 2 — Figure S1 Comparison of the behaviour of the 91 connections in raw and COMMIT‐weighted connectomes for both healthy controls (HC) and progressive multiple sclerosis patients (PMS). The overall behaviour of the connections’ strength shows a high correlation between the methods but looking at individual connections we see how COMMIT is able to enlarge or shrink the differences between the two groups of subjects. [file HBM-41-2951-s002.png]
